# Chicago Medical Society

**Published:** 1875-03-01

**Authors:** 


### CHICAGO MEDICAL SOCIETY.

### Regular Meeting, January 4th, 1875.

DR. LEE, Chairman of the Committee on Surgery, read a paper on Anæsthetics, and exhibited an Ether Inhaler, invented by Dr. Morgan, of Dublin. The doctor referred briefly to the discovery of ether and chloroform as anæsthetic agents, and gave some results of the investigations of a committee appointed in England some years ago, to report on the relative merits of the two agents. The primary action of both is stimulating; the secondary, depressing — ether stimulates the respiratory, chloroform the circulatory functions. Ether is safer, though slower in its action. Out of 92,815 administrations of ether, there were 4 deaths. From 152,000 administrations of chloroform, there were 84 deaths; while in 5,588 inhalations of a mixture of the two, there was one death.

Dr. Andrews, of this city, had reported that chloroform was eight times more dangerous than ether, and twice as dangerous as a mixture of chloroform and ether.

In the use of ether, the stages of anæsthesia are better marked; the patient coming more gradually under its influence, giving a better opportunity to observe the symptoms of danger. The manner of administering has much to do with the safety of the agents. American surgeons advocate the use of ether more now than formerly, though many still advocate the use of chloroform, believing it to be safer and better for the patient, claiming that there is only danger in certain cases owing to idiosyncrasy and careless administration. In illustration of the peculiar effects in certain cases, a case was related in the doctor's own experience. The patient was to be operated on for Strabismus. Chloroform was inhaled, when the heart's action became so violent, he had to desist. About a year afterwards, ether was administered, and the patient came readily under its influence without any untoward symptoms. In cases of nervous shock, ether stimulates when properly used, and do not believe it can be used too soon in these cases.

Chloroform is better borne than ether by patients under five years old. Once administered chloroform to a young woman in confinement, at intervals, for five hours, with good results. Years afterwards, had occasion to use it again for purposes of extracting a needle from this same patient. Respiration became suspended, and it was with difficulty reestablished.

Ether had been used for a long time in the north of Ireland, as an intoxicating agent—from two to four drachms being taken at a time, two, four, or six times daily. It acts very much like alcohol; thinks it is eliminated by the lungs: does not cause any peculiar evils, and is less dangerous than whisky. Three gallons of ether has the intoxicating effect of ten gallons of whisky.

Here the doctor exhibited the inhaler, and explained its use and advantages. It is a tin can with an elastic diaphragm. A flexible tube connects the can with the mouthpiece, the aperture of which is surrounded by an elastic air-cushion, which prevents the patient from breathing any air, except what may be in the can with the ether.

Has used the inhaler some twenty times, and claims for it besides perfect safety, two great advantages:

1st. The ease and rapidity with which anæsthesia can be produced—the limits of time being one and a half minutes and ten minutes; average time, about four minutes.

2d. The small quantity of ether required; a few drachms generally being sufficient.

Was at Mercy Hospital when one pound of ether had been used without effect. The inhaler was applied, and one ounce of ether produced insensibility.

In the discussion of the paper, Dr. T. D. Fitch said he was much interested in the subject. He believed that death always commenced at the lungs, as the heart generally beats fifteen or twenty minutes after respiration stopped. While in the army, and for a long time after, he was a firm believer in chloroform—believed it safer and better in the long run than ether. Changed his opinion since he lost a patient some five years ago. There is less immediate danger in ether. Thought the reason why chloroform was not dangerous in parturition, was owing to the fact that pain was present, and that the patient was not frightened. One objection against ether, however, is the poisonous effect on the blood, as sometimes shown in slow recoveries after its use.

Dr. C. M. Fitch asked, if it was not probable that the fatal result in Dr. T. D. Fitch's case, was due to paralysis of the pneumogastric nerve, death not beginning at either lungs or heart? He knew patients could be restored where death was imminent from use of chloroform, by suspending them with head downwards. Mice could be restored by suspending them by the tail.

Dr. Paoli had used ether in two cases of labor, in both of which the pains were suspended; now uses chloral hydrate in forceps cases. It renders patient insensible, but does not suspend labor.

Dr. Quine considered there was a radical difference in the mode of operation of the two agents.

Dr. Ingals related a case of a death from chloroform which occurred to him. An apparently strong, healthy woman, struggled during the administration. The pulse ceased before respiration. Post-mortem showed a fatty heart.

Dr. T. D Fitch asked for the experience of members present in the use of anæsthetics in Parturition. Do they hasten or retard labor? He considers that they always diminish the pains, and that after the expulsion of the child, the uterus does not contract so well, they rendering the patient liable to hæmorrhage.

Dr. Adolphus never uses them—always gives morphia when there is need of anything which increases the contractions of the uterus, diminishing the irritability, rendering the pains more efficient.

Dr. Graham exhibited to the Society, two kidneys taken from a subject in the dissecting room. The right one was very much enlarged. In the left, there was almost complete atrophy of the parenchymatous substance; and instead, there were several large pockets distended with purulent fluid. In one of the pockets there was a small calculus, adherent to the mucous membrane; and in the pelvis of the organ, there was a large calculus, with a process extending into the ureter, completely occluding it.

Dr. Quine had seen the patient before death at the hospital, and he was asked to give the history of the case as far as he knew it. He stated that he had seen the patient but once, and she was then moribund from puerperal fever. She had been confined in the hospital; but since her admission, there had been no symptoms of any renal disease. At the autopsy, there was found extensive peritonitis. The pericardium was found distended with fluid. Purulent fluid was also found in the pleural cavities. The immediate cause of death was apparent, and for that reason the kidneys had not been examined.

